# We'll Meet Again: Revealing Distributional and Temporal Patterns of Social Contact

**DOI:** 10.1371/journal.pone.0086081

**Published:** 2014-01-27

**Authors:** Thorsten Pachur, Lael J. Schooler, Jeffrey R. Stevens

**Affiliations:** 1 Center for Adaptive Rationality, Max Planck Institute for Human Development, Berlin, Germany; 2 Center for Adaptive Behavior and Cognition, Max Planck Institute for Human Development, Berlin, Germany; 3 Department of Psychology, University of Nebraska-Lincoln, Lincoln, Nebraska, United States of America; Institut Pluridisciplinaire Hubert Curien, France

## Abstract

What are the dynamics and regularities underlying social contact, and how can contact with the people in one's social network be predicted? In order to characterize distributional and temporal patterns underlying contact probability, we asked 40 participants to keep a diary of their social contacts for 100 consecutive days. Using a memory framework previously used to study environmental regularities, we predicted that the probability of future contact would follow in systematic ways from the frequency, recency, and spacing of previous contact. The distribution of contact probability across the members of a person's social network was highly skewed, following an exponential function. As predicted, it emerged that future contact scaled linearly with frequency of past contact, proportionally to a power function with recency of past contact, and differentially according to the spacing of past contact. These relations emerged across different contact media and irrespective of whether the participant initiated or received contact. We discuss how the identification of these regularities might inspire more realistic analyses of behavior in social networks (e.g., attitude formation, cooperation).

## Introduction

On 17 March 2009, doctors detected the first human case of H1N1 influenza on a Mexican farm. H1N1 quickly spread worldwide and by the end of 2009, the World Health Organization estimated over 600,000 cases in 208 countries, accounting for at least 12,000 deaths [1], [2]. Disease transmission, a vital issue in today's highly connected society, offers an important application for studying social networks. For instance, it has been shown that the addition of even a small proportion of random connections among network nodes can dramatically lower the critical level of infectiousness above which a disease turns into an epidemic [3]. Social networks have been investigated by social scientists for over 50 years; they have recently also attracted the interest of physicists and biologists [4], [5]. Most analyses have focused on the *structure* (or topology) of a network, such as the number of partners with which individuals interact (degree), the interconnections among individuals (clustering), and the number of connections required to connect two individuals (path length). Relatively little work has examined *connection strengths* (edge weights), that is, the intensity of interactions between network members. In their pioneering research, de Sola Pool and Kochen [6] acknowledged that not all connections in a social network are equally strong. For simplicity's sake, however, they assumed equal strength in their analyses, and subsequent work on social networks paid little heed to the strength of connections.

The goal of this article is to study regularities in connection strength, defined as the probability of social contact of an individual to the different members of her social network, by (a) examining the distribution of contact probability across network members and (b) predicting future contact probability based on aspects of previous contact. Specifically, we demonstrate how predictions for regularities in the dynamics of contact probability can be derived from a memory model that has previously been used to identify environmental regularities (e.g., in word use). This model predicts that future contact follows from the frequency, recency, and spacing of previous contacts in very specific ways.

To examine connection strength, we conducted a diary study in which participants recorded, over an extended period of time, contacts with members of their social networks. Our analysis of the interaction patterns underlying social contact enables more realistic models both of social systems and of cognition at the level of the individual. In the Discussion section, we elaborate how regularities in social contact are important in attitude formation [7] and social transmission, and how they can be critical in the evolution of cooperation [8]; moreover, we discuss how they are related to functional accounts of memory [9].

### Distribution of Social Contact

Although network analyses often focus exclusively on the presence versus absence of a connection, several researchers have highlighted that differences in connection strength (i.e., weighted networks) can be critical for understanding social networks. Granovetter [10], for instance, distinguished between “weak” and “strong” connections, arguing that the former in particular drive the surprising interconnectedness of social systems. In analyses of networks among scientists, Newman [11] has shown that patterns of scientific collaborations are better captured when differential connection strengths are allowed. Both Yan, Zhou, Wang, Fu, and Wang [12] and Read, Eames, and Edmunds [13] have demonstrated in simulations that infectious diseases spread more slowly in weighted than in unweighted networks. In addition, many theoretical studies have emphasized the importance of connection strength in social networks [11], [14]–[16]. Against this background, it seems surprising that few empirical data exist on how the connection strengths of a person to members of her social network (e.g., operationalized as the probability of contact) are typically distributed.

Research on social relations has shown a strongly differentiated structure in social connections. For instance, people are known to have very “close” contact with just a few members of their social network [17]–[19]. However, precise quantifications of how connection strength is distributed across a person's network members are rare. Are values normally distributed around a “typical” contact probability with only rare extreme values; or is the distribution skewed, with very few high probabilities and very many low probabilities (or vice versa)? In one of the few studies examining the distribution of connection strengths, Ramasco and Gonçalvez [20] observed a power distribution (of collaboration probability) in a network of movie actors, indicating a highly skewed distribution. We aim to examine the empirical distribution of connection strength in day-to-day interactions.

### Temporal Dynamics of Social Contact: How Does The Past Predict the Future?

Another important issue for understanding connection dynamics in social networks is how contact probability with an individual network member can be predicted [16]. A network member's contact probability is predictable to the extent that it follows certain regularities; we are not aware of any analyses that have systematically examined such regularities. To explore the temporal dynamics of social contact, we use the theoretical framework of the rational analysis of memory [9], which ties memory performance to the statistical structure of the environment. Our analyses are based on a model that assumes that memory phenomena—in particular, learning curves, forgetting curves, and spacing effects—represent efficient responses to typical patterns of occurrence in the environment. Anderson and Schooler [21] found evidence for regularities in the relationship between the probability of a certain word's occurrence and the pattern of previous uses of that word. The results of another analysis by Anderson and Schooler hinted that systematic relations might also exist in social contacts. Analyzing three years' worth of email received by John Anderson (JA), they observed three patterns relating the probability of future email contact to the frequency, recency, and spacing of previous contact, respectively. We next describe these regularities in more detail.

#### Frequency effects

The probability *p* of someone contacting JA on day *t* increased linearly with the number of days *f* on which he had contact with this person in the previous *w* days: *p* = *b_0_*+*b_1_f*. In this equation, the intercept *b_0_* represents the probability of encountering a network member given that one has not encountered her in the window of interest, and *b_1_* reflects the degree to which the probability increases with more frequent previous contacts. Thus, the probability of future contact increases linearly with the frequency of previous contacts.

#### Recency effects

Anderson and Schooler [21] also found that the recency or time since last contact was strongly related to the probability of future contact. Specifically, the relation followed a power function between the odds *o = p/*(1-*p*) that someone contacted JA and the number of days *r* since he last had contact with that person (on day *t*-*r*): *o* = *cr* ^−*α*^. The parameter *c* represents the odds of contact today after the last contact being one day ago, and α reflects how quickly the odds of contact decrease as the number of days since the last contact increases. The power function implies a scale-free relationship; that is, the odds of contact show a similar pattern independent of whether recency is scaled in minutes, days, or years.

#### Spacing effects

Finally, Anderson and Schooler [21] found evidence that future contact is also affected by the spacing of past contacts. Take two persons with whom JA had the same number of contacts previously, but for one person the contacts were spread out over time (*spaced contacts*), whereas for the other person the contacts occurred clustered together in time (*massed contacts*). The probability of contact soon after the last contact was higher for the massed contacts, whereas the pattern was reversed for the probability of contact at longer lags.

Anderson and Schooler's [21] single-case study based on email contacts hints that social interactions might follow certain regularities. Moreover, it suggests that an approach originally proposed to study memory offers a useful tool to explore dynamics in social contact. Our diary study reported below extends Anderson and Schooler's work in a number of important ways. First, rather than focusing on a single person, our study involves a group of 40 participants. Second, our analysis tracks not only received email correspondence, but both received and self-initiated contacts in face-to-face interactions, phone calls, and other contact media. Finally, Anderson and Schooler collected their data in the early days of email. Even with the rise of email use, for heavy users email communication comprises only about 25% of social contact [22]. As a result, it is currently unclear whether the regularities Anderson and Schooler observed can be found in social contact more generally.

In sum, whereas empirical and theoretical work on social networks has previously focused on the static structure of social networks, we emphasize the dynamics of social contact. Next, we report a study exploring regularities in the distribution of social contact and in the relationships between past and future contact based on people's day-to-day encounters.

## Methods

### Ethics Statement

The study was approved by the Ethics Committee at the Max Planck Institute for Human Development. Participants completed a written informed consent form before starting the study.

### Participants, Materials, and Procedure

We recruited 40 participants (20 male, 20 female, mean age 25.2 years; range 19–31) from universities in Berlin and paid them €100 each for taking part in the study. They were instructed to record their daily social contacts in a diary for 100 consecutive days. To ensure that the recorded contacts met a minimum level of relevance, we defined social contact as all face-to-face or phone conversations lasting at least 5 minutes and all communication conveyed electronically or on paper of at least 100 words in length. The diary was a booklet containing a matrix, with rows representing individuals with whom contact occurred and columns representing days. Participants added the names of the individuals with whom social contact occurred to the rows after the first contact and then entered all ensuing contacts with that person in that row. For each day on which contact with a network member occurred, participants entered into the day's cell the contact mode (face-to-face, email/letter, phone, or other) and the direction of the contact, defined as whether the contact occurred on the other person's initiative or their own. If there were multiple contacts with a network member on a given day, participants recorded only the first contact. To decrease the risk of data loss, we encouraged participants to enter data by the end of each day at the latest. After completing the diary, participants classified each network member listed to one of eight categories (romantic partner, friend, flatmate, family, work colleague, acquaintance, relative, or other). To ensure anonymity, participants erased the names of the contacts from the diary before mailing it back to the experimenter. The raw data are available in File S1 and from the Dryad Digital Repository: http://doi.org/10.5061/dryad.pc54g.

## Results

### Distribution of Social Contact

**Figure 1:**
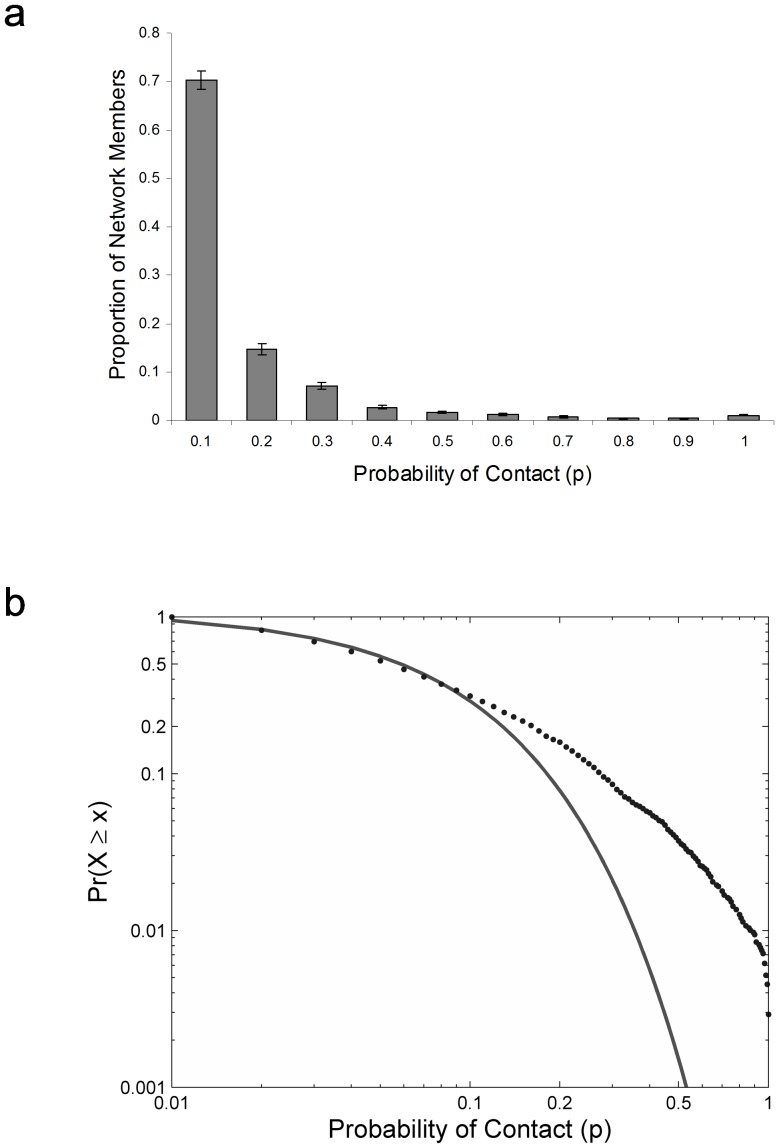
Distribution of contacts across network members. (a) The mean (across participants) proportion of network members with contact probability *p*, binned for 10 levels of *p* (i.e., the first bin contains network members with *p* = 0.01–0.1, the second bin those with *p* = 0.101–0.2, etc.). Error bars represent standard errors of the mean. (b) Cumulative distribution of the collapsed individual data in a log-log plot, showing for different levels of contact probability *p* the proportion of participants with that contact probability or higher. This distribution is well captured by an exponential function.

Over the 100 days, each participant recorded contacts with, on average, 77.1 (*SD* = 33.2; range 26–155) different network members, with *M* = 8.3 (*SD* = 3.77; range 2.3–18.9) contacts per day. To determine the distribution of connection strength across the different network members, we calculated for each her contact probability across the 100 days, defined as the proportion of days on which a contact occurred. Figure 1a shows for 10 binned levels of contact probability the average (across participants) proportion of network members that fell into the respective bin. The distribution was highly skewed, with a small fraction of a person's network accounting for a large share of contacts. The median contact probability with any network member across participants was 5.5%. [Note that the lower a network member's contact probability, the lower her chance of being recorded in a diary covering a limited amount of time. With longer study periods, an even lower median contact probability can be expected [23]). Contact probability was 50% or higher for only 3.8% of participants' network members; and 20% of the network members accounted for, on average, 63.4% of the social contacts. Note that because we disregarded multiple contacts with the same person on one day, our data may underestimate the skewness (and predictability) of social contact. How were contacts distributed across the different social categories of network members? Although close network members (i.e., romantic partner, friends, family, relatives, and flatmates) constituted the minority of the participants' social networks (38%), this group was involved in the majority of the contacts (68%).

Figure 1b plots the individual data (collapsed across participants) as a cumulative distribution function in a log-log plot. That is, the figure shows for the different levels of contact probability *p* the proportion of network members with contact probability *p* or higher. The distribution is well captured by an exponential function, *Pr*(*P*≥*p*) = 1.093 *e* ^−13.25 *p*^ (*R^2^* = 0.986). An exponential function fitted the data better than a power function *Pr*(*P*≥*p*) = 0.109 *p* ^−0.494^ (*R^2^* = 0.458). Moreover, as shown in Figure S1, when the individual data were fit for each participant separately, an exponential function yielded a better fit for 31 of the 40 participants (77.5%). Note that in a log-log scale, an exponential function curves down, whereas a power function results in a linear relationship. An exponential function results in a linear relationship in a log-linear scale. In sum, our data suggest that frequent contact occurs with only a very small number of network members, and that with most members contact is relatively rare.

### Temporal Patterns of Contact

Next, we turn to the relationship between the probability of future contact and aspects of past contact. Specifically, we examine whether the probability of contact on day *w*+1 can be predicted by the frequency, recency, and spacing of contact in a window of *w* days. For all analyses, *w* was set to 30. We chose this window size because it strikes a good balance between being sufficiently large for a relationship to manifest itself and allowing for a substantial number of windows that can be calculated across the study period of 100 days. Analyses using alternative window sizes (see Figure S2 and Table S1) led to similar conclusions. (For alternative analyses, predicting the probability of having at least one contact between day *w*+1 and day 100, see Figure S3 and Table S2.)

#### Frequency effects

**Figure 2:**
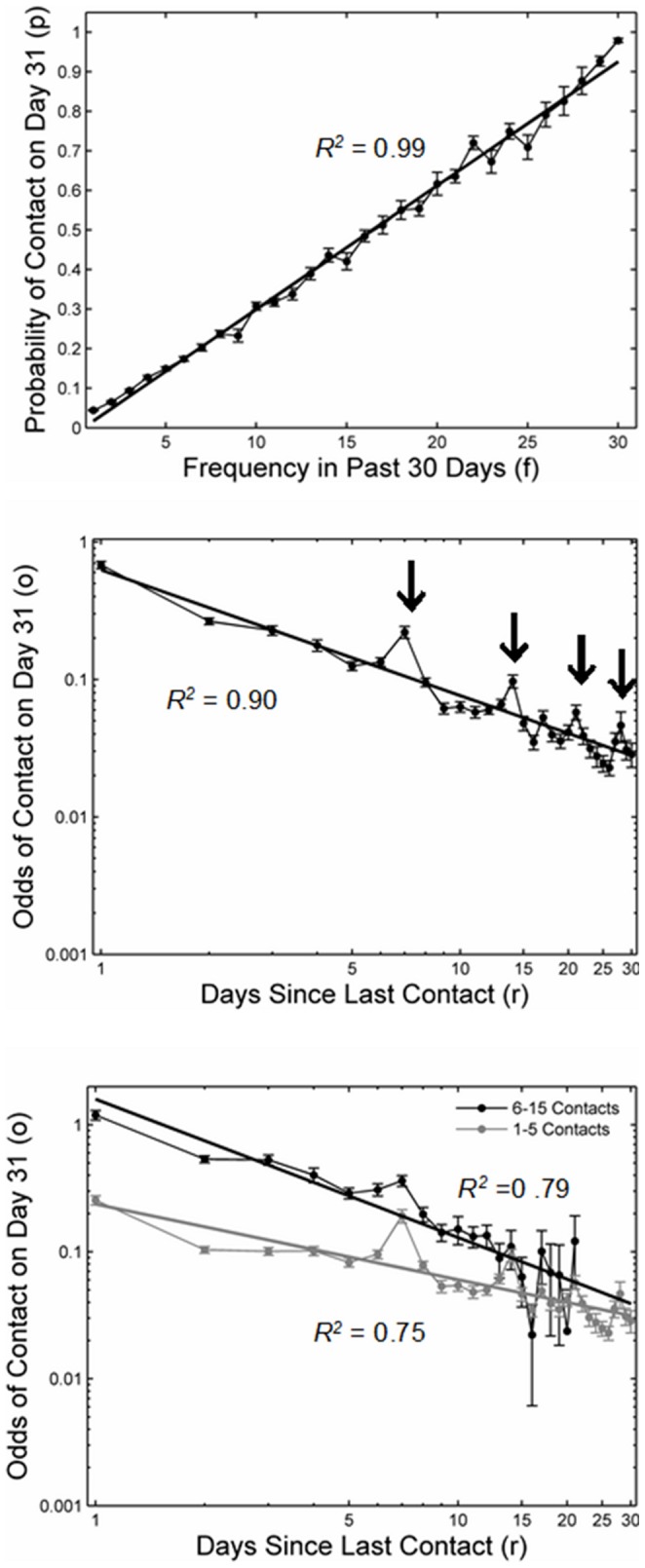
Regularities in social contact. (a) Probability of contact *p* increases linearly with the frequency of past contacts *f*. (b) The odds of contact are a power function of the number of days since the last contact, appearing as a straight line on the log-log plot. (c) The power relationship holds for both low- and high-frequency contacts. Shown are bootstrapping standard errors calculated using the normal approximation method. For the bootstrapping analysis, data from 40 participants were drawn (with replacement) from our sample and aggregated across participants. We repeated this procedure 1,000 times. *R^2^* values in (b) and (c) were computed on the log-transformed data.

We calculated the probability of contact with a person on the 31^st^ day as a function of the number of contacts *f* with that person in the preceding 30 days. Figure 2a indicates a strong linear relationship, with the probability of contact increasing proportionally with the frequency of previous contacts, *p* = −0.01+0.03 *f* (*R^2^* = 0.99).

#### Recency effects

For various values of *r* (i.e., the number of days since last contact), we determined those network members with whom the last contact occurred exactly *r* days ago in the time window of 30 days and calculated the proportion of network members for whom a contact occurred on the 31^st^ day. Figure 2b shows the odds of contact as a function of the recency of the last contact plotted on a log-log scale. (As power functions are unbounded above, *Y* cannot be a bounded measure, such as probability of contact. Therefore, we instead used odds, which—like power functions—are unbounded above.) As can be seen, the data are well described by the power function *o* = 0.63 *r* ^−*0.91*^ (*R^2^* = 0.90). Figure 2b also reveals some systematic deviations from a power relationship, however, with the probability of contact spiking at *r* = 7, 14, 21, and 28. These spikes are likely to reflect another regularity, namely weekly cycles in social activity.

#### Combined frequency and recency effects

Frequency typically correlates with recency of contact. That is, the last contact with a person encountered frequently will tend to have occurred more recently than the last contact with a person encountered only rarely. To compute this correlation, we determined for each time a contact to someone occurred on day 31, the number of days since the last contact as well as the number of contacts to that person in the previous 30 days. In our data, this (Pearson) correlation was *r* = −0.60 (*p* = 0.001). To test whether the power function between the odds of contact and the recency of the last contact also held when frequency was kept constant, we analyzed the relation separately for different levels of contact frequency. Figure 2c shows the odds of probability of contact as a function of recency for network members with high contact frequency (defined as 6–15 contacts in the previous 30 days) and those with low contact frequency (1–5 contacts). As can be seen, the patterns are rather similar for both levels of contact frequency, implying that the relationship between contact probability and recency holds irrespective of frequency. The best fitting functions were *o* = 1.57 *r* ^−1.08^ (*R^2^* = 0.79) and *o* = 0.24 *r* ^−0.60^ (*R^2^* = 0.77) for high and low contact frequencies, respectively.

#### Spacing effects

**Figure 3:**
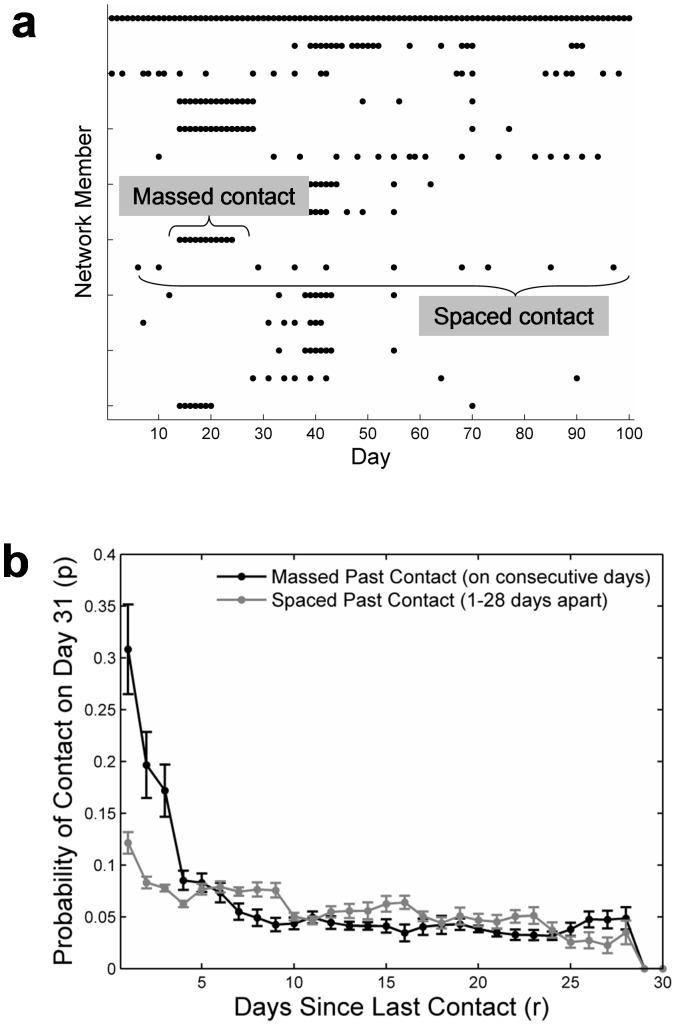
Spaced and massed social contact. (a) An example for a participant's pattern of social contacts with her network members across a period of 100 days, focusing on the participant's 15 network members with the highest contact probability. The distribution of contacts across time varies among network members, some showing a spaced pattern and others showing a clumped or massed pattern. (b) The probability of contact *p* (when *f* is held constant at 2) depends on both the number of days since the last contact and the spacing of the past contact. The data points for days 3 through 28 are running averages over 5-day bins, including data from the two preceding and the two subsequent days (e.g., the running average for day 3 was computed as the average over the days 1–5). Days 2 and 29 are running averages over 3-day bins, and days 1 and 30 are moving windows over 1-day bins. Shown are bootstrapping standard errors (determined using the normal approximation method).

Figure 3a shows the pattern of contacts for a typical participant. As can be seen, contacts with some network members occurred in clusters across the 100 days, whereas contacts with others were spaced relatively equally. To examine how the spacing of past contact affected the probability of future contact, we selected from all participants cases with exactly two contacts (i.e., *f* = 2) in the past 30 days and determined the probability of contact with these cases on the 31^st^ day. (Similar results were obtained for higher values of *f*.) Massed contacts were defined as cases in which the two contacts occurred on two consecutive days; spaced contacts, as cases with a lag of 1 to 28 days intervening between the two contacts. Figure 3b plots the probability of contact as a function of recency, separately for massed and spaced contacts. As indicated by the intersection of the lines for massed and spaced contacts in the figure, there was an interaction between recency and the distribution of past contacts over time. More specifically, at short recencies (i.e., briefly after the last contact), the probability of contact was higher for massed than for spaced contacts. At longer recencies (i.e., when the last contact occurred some time ago), however, the pattern was reversed. The decay parameter *α*, indicating how quickly the odds of contact decrease as the number of days since the last contact increases, was higher for massed than for spaced contacts (.702 vs. .360). These results suggest that taking into account the spacing of past contacts helps to predict the probability of future contact.

### Contact Direction and Medium

**Figure 4:**
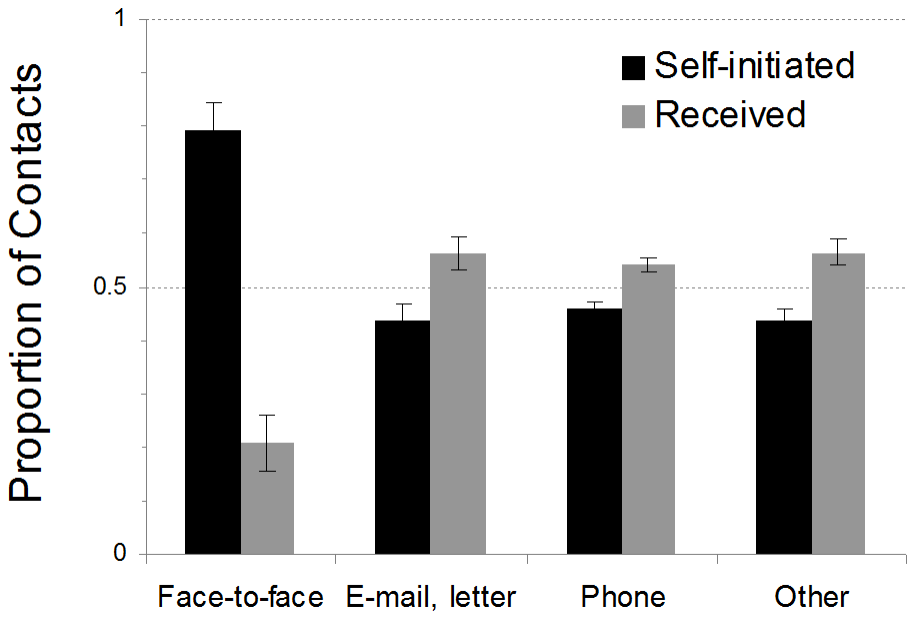
Proportion of recorded contacts as a function of contact medium and direction. Error bars represent standard errors of the mean.

Do the regularities in social contact depend on whether the contact was recorded as received or self-initiated, and/or on the contact medium? Participants indicated that they had initiated 69.9% of the recorded contacts. As each contact has both a receiver and a sender, one might have expected equal numbers of self-initiated and received contacts in the world. Possible reasons for the data departing from such an equal distribution might be that participants were somewhat more likely to forget received contacts, or that they tended to code contacts with an ambiguous direction as self-initiated. Alternatively, people who take initiative might be overrepresented in our sample. The majority of recorded contacts were spoken and simultaneous communications (face-to-face: 66.1%; phone: 17.1%); written communications occurred less frequently (email and letters: 8.1%). Interestingly, Figure 4 suggests an interaction between direction and contact medium: 80% of face-to-face contacts were recorded as being self-initiated, whereas the direction of contact for the other types of contact media was more symmetrical, with a slight bias towards received contact.

**Figure 5:**
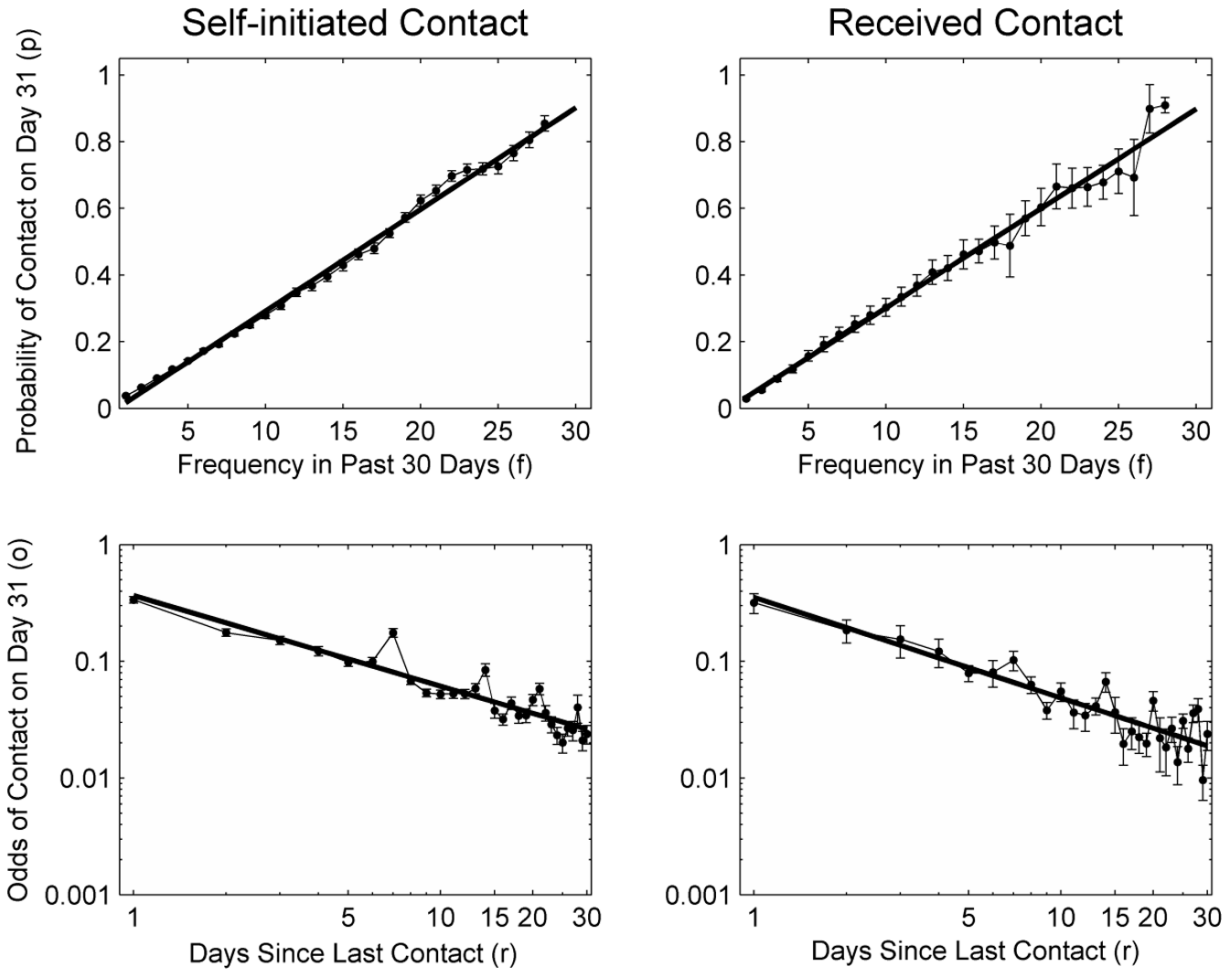
Direction-of-contact effects on frequency and recency. For the frequency analysis (upper row), the data points for frequency 3 through 28 are running averages over five frequency level bins, including data from the two preceding and the two subsequent frequency levels. The frequency levels 2 and 29 are running averages over three frequency level bins, and frequency levels 1 and 30 are moving windows over one frequency level bin. Shown are bootstrapping standard errors (determined using the normal approximation method).

Importantly, Figure 5 shows that the patterns in the relation between contact probability and frequency and recency held for both self-initiated and received contact (see Table S3 for regression functions). The only qualification is that the 7-day spikes were less pronounced for received contact. Given the overall robustness of the patterns, it seems unlikely that the differences in the distribution of received and self-initiated contact, possible biases in the reporting of contacts, or an overrepresentation of proactive people in our sample critically biased our conclusions.

**Figure 6:**
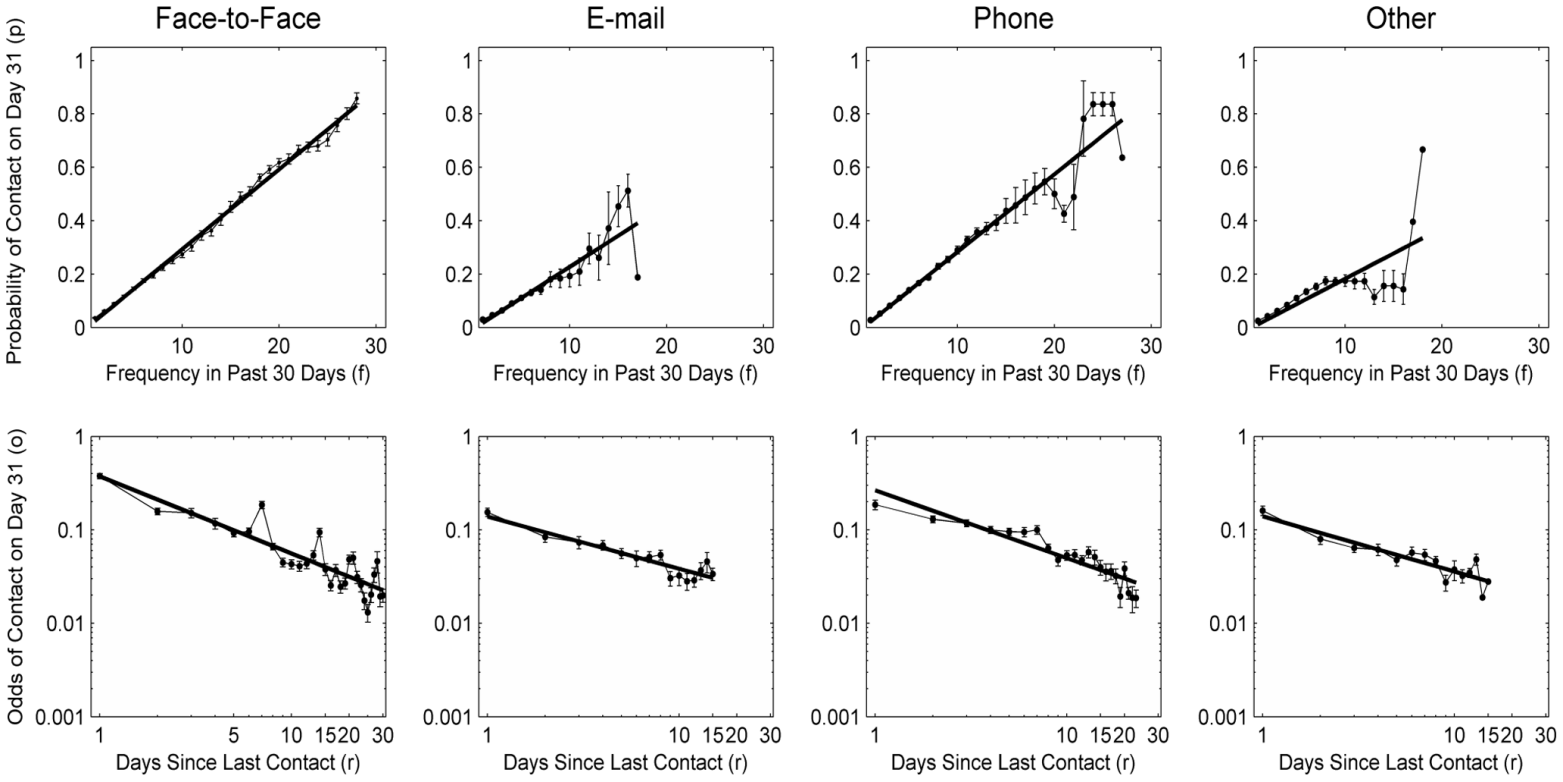
Contact-medium effects on frequency and recency. For the frequency analysis (upper row), the data points are binned as described for Figure 5. Shown are bootstrapping standard errors (determined using the normal approximation method).

As can be seen in Figure 6, the effects of frequency and recency also seem to hold across the different types of contact media (see Table S4 for regression functions). However, the figure also shows that for email contacts, more past contacts did not increase the probability of future contact as strongly as they did for face-to-face or phone communication. This may result from the indirect and asynchronous nature of email communication. Moreover, the 7-day spikes in the analyses of recency (lower graphs) were more pronounced for face-to-face interactions than for the other contact media.

## Discussion

As a rich source of both important opportunities and information, the social environment represents a key contextual variable for the human species. Our analyses show that recording social contacts over a little more than three months allows one to make pretty good bets about a person's contacts on any given day. Two main findings emerged. First, in contrast to the frequent assumption in theoretical analyses of cooperation that the distribution of contact probability is equal across network members, we found a highly skewed distribution that is well described by an exponential function (Figure 1b). This finding indicates that spread of contagions such as fads or viruses is more likely to occur via one's family than via acquaintances. Second, the probability of future contact follows the temporal dynamics of past social contacts in highly specific ways. Namely, future contact scales linearly with frequency of past contact, proportionally to a power function with recency of past contact, and differentially according to the spacing of past contact. Moreover, in the aggregate, contact probability displays patterns of weekly cycles. Whereas these cycles emerged both for self-initiated and received contacts, they were most pronounced in face-to-face contacts, much attenuated in telephone contacts, and barely apparent in email contacts. Weekly cycles have also been found in recency curves based on where and when people drive (Schooler, unpublished data), suggesting that moving about in the world may explain why weekly cycles are more pronounced for face-to-face contact than for the other types of contact media. Overall, caution is thus warranted in generalizing from one form of social contact to another. For instance, the statistics of contacting friends on Facebook need not reflect the statistics of face-to-face contact with friends.

Our findings have a number of methodological and conceptual implications for (a) the use of email contact as a proxy for social contact more generally, (b) models of attitude formation, (c) models of social transmission, (d) models of the evolution of cooperation, and (e) the rational analysis of memory. We next describe each of these implications in turn.

Analyses of email communication have been critical for the development and testing of recent models of social networks [24], [25]. Our finding that patterns of contacts via common contact media, such as face-to-face contacts, parallel those found for email indicates that the results of email-based analyses (e.g., concerning the evolution of networks) may hold for social contact more generally. As noted above, one exception is that weekly spikes of contact seem to be less pronounced in email than in face-to-face contact.

Social contact provides an opportunity for social influence. Importantly, models of attitude formation often include frequency of contact as one key variable influencing how attitudes change. For instance, according to Latané's [7] social impact theory, the social impact emanating from a network member is a function of her persuasiveness and immediacy to the target person, with immediacy often defined as probability of contact [26]. In a computer simulation implementing social impact theory, Nowak et al. examined the dynamics of attitude formation over time. They represented a population of people as cells in a quadratic grid, and probability of contact was operationalized as the distance between the cells in this grid. Whereas in these analyses the distribution of contact probability was superimposed by the distribution of distances in the grid, our results give an indication of the shape of the distribution in actual social networks. Moreover, our results allow an implementation of contact probabilities that vary over time in a realistic fashion, subject to the regularities we identified, rather than having to assume fixed contact probabilities.

Social contact also provides an opportunity for transmission of fads, rumors, and infections. Mathematical epidemiologists have studied the speed and nature of the propagation of disease in a social network [27], [28] and found that network properties such as clustering and degree distribution [29], [30] and, more recently, connection strength [13] strongly predict the resulting propagation pattern. Our finding that the distribution of contact probability is considerably skewed may lead to novel predictions in the study of social transmission. For instance, whereas it is usually argued that propagation in a network can be fast and wide due to the existence of “weak,” random connections [3], [31], our results indicate that these connections in fact tend to be used very infrequently. As a consequence, networks might prove to be rather robust against viral spread, even when the proportion of random connections is quite high.

Further, social contact provides an opportunity for cooperative interactions, in which partners decide whether to help the other or to be selfish [32]. Importantly, the probability of future contact, *p*—or as Axelrod [33] dubbed it, the “shadow of the future”—is a key variable for the evolution of some strategies for cooperation. For instance, take the tit-for-tat strategy (TFT; [34]), which starts by cooperating, and then simply copies the partner's previous choice. TFT has been shown to outperform purely selfish behavior as long as *p*>*c*/*b*, where *c* represents the cost of cooperating and *b* represents the benefit from receiving the altruistic act [8], [34]. With a probability of contact as low as the median of 5.5% that we observed, the benefits of cooperation need to exceed the costs by a ratio of at least 18∶1 before TFT can evolve. (Specifically, solving *p*>*c/b* with *p* = 0.05 yields 0.05>1/18.2). Though such high benefit-to-cost ratios may hold in some cooperative situations, such as food sharing or predator alarm calls, it is not clear how frequently they occur in the real world.

Moreover, note that analyses of the necessary benefit-to-cost ratio are based on the standard assumption in evolutionary game theory that every player has an equal chance to play against other players [35]. Our results clearly contradict this assumption. As discussed in Pachur, Schooler, and Stevens [23], TFT (and, by extension, other strategies that depend on repeated interactions) might evolve very differently under more realistic circumstances. Specifically, a potentially viable modification of TFT may attend to the probability of future contact and implement TFT only with partners likely to be encountered frequently [23]. This requires tracking estimates of future contact. If these estimates involve uncertainty, however, it remains possible that cooperation may still evolve, even with low probability of future contact. This can occur when costs of the error of defecting against a partner with high probability of future encounter may outweigh costs of the error of cooperating in a true one-shot game [36]. In addition, the regression equation for frequency effects predicts a nonzero probability of future contact after a single interaction in the last 30 days (this holds for window sizes up to 90 days; see Table A4). This finding supports the notion that true one-shot interactions are rare and that the shadow of the future remains pervasive [32]. In short, given the central role of the probability of future contact for the evolution of cooperation, insights into the distribution of contact probability in actual social networks can have considerable implications for the conditions under which cooperative strategies emerge.

If the probability of future contact is an important determinant of (cooperative) behavior, how could the cognitive system estimate this probability? As proposed by Pachur et al. [23], one possibility is to exploit activation in memory. The rational analysis of memory [9] assumes that human memory makes information available as a function of how likely it is to be relevant in the current context. In ACT-R [37], which incorporates the rational analysis of memory, the accessibility of a memory record is determined by its activation. This activation, in turn, is a function of the probability *p* that the record will be needed to achieve a processing goal (“need probability”). In the analyses reported here, the *p* of needing information about a person in one's social network represents the probability of encountering that person. Given the link between past and future contact, current activation may not only indicate whether a memory is immediately relevant, but additionally predict future activation (i.e., the probability of seeing a person again). Assuming that activation in memory translates into higher familiarity, social heuristics might therefore exploit the statistical structure of social contacts simply via a sense of familiarity. In addition, an understanding of the mechanisms underlying social memory is also relevant for decision making beyond cooperation decisions [38]. After all, the mind often seems to recruit information about the social environment to make inferences about the world in general [39], [40].

## Conclusions

If our social world were structured in the way that traditional models of social networks assume, our lives would appear most odd. On any given day, we would be as likely to catch a cold from our local butcher as from our children. These models put aside the complexities of our social world: the daily routines of work and leisure, the unexpected encounters with friends in the check-out line, or the weekly poker game. Yet our analyses show that these complexities conspire to yield remarkably simple and predictable patterns of social contact that can support more realistic theorizing about and simulation of our social world.

## Supporting Information

Figure S1 **Individual Fits of Exponential and Power Functions on the Distribution of Contact Probability.** Both exponential and power functions were fitted to the individual distributions of contact probability across the members of the person's social network. For each participant the distribution and the best-fitting function is shown on a log-log scale. For 31 of the 40 participants, an exponential function (curved relationship) yielded a better fit than a power function (linear relationship).
(TIF)

Figure S2 **Frequency, Recency, and Spacing Effects on Contact Probability for Window Sizes** ***w*** **= 10, 30, 50, 70, and 90.** In the recency analysis separating frequent and rare contacts, frequent contacts were defined as those occurring on at least one fifth of the days in the time window (that is, on ≥*w*/5 days); rare contacts were defined as those occurring less frequently than that. *R^2^* values in the second and third rows were computed on the log-transformed data.
(TIF)

Figure S3 **Frequency, Recency, and Spacing Effects on Presence Probability for Window Sizes** ***w*** **= 10, 30, 50, 70, and 90.** Presence probability is defined as the probability that there is at least one contact during days w+1 to 100. In the recency analysis separating frequent and rare contacts, frequent contacts were defined as those occurring on at least one fifth of the days in the time window (that is, on ≥*w*/5 days); rare contacts were defined as those occurring less frequently than that. *R^2^* values in the second and third rows were computed on the log-transformed data. *R^2^* values in the second and third rows were computed on the log-transformed data.
(TIF)

File S1 **Raw Data.**
(ZIP)

Table S1 **Regression functions for frequency and recency effects when using window sizes 10, 30, 50, 70, and 90.**
(DOCX)

Table S2 **As a focus on the probability of contact on a particular day (i.e.,** ***w*** **+1) might be overly restrictive, we also examined how the frequency and recency of contact in the time window of size** ***w*** **relates to the probability of another contact on at least one of the remaining days (i.e., day** ***w*** **+1 to day 100), to which we refer to as ‘presence probability’.** (For the effect of the spacing of contacts on presence probability, see Figure S3).
(DOCX)

Table S3 **Regression functions for frequency and recency effects separately for self-initiated and received contacts.**
(DOCX)

Table S4 **Regression functions for frequency and recency effects on contact medium.**
(DOCX)
