# Supplementary figures and images for: We'll Meet Again: Revealing Distributional and Temporal Patterns of Social Contact
Source: PLoS One. 2014 Jan 27;9(1):e86081. doi: 10.1371/journal.pone.0086081 (PMC3903503; doi:10.1371/journal.pone.0086081)

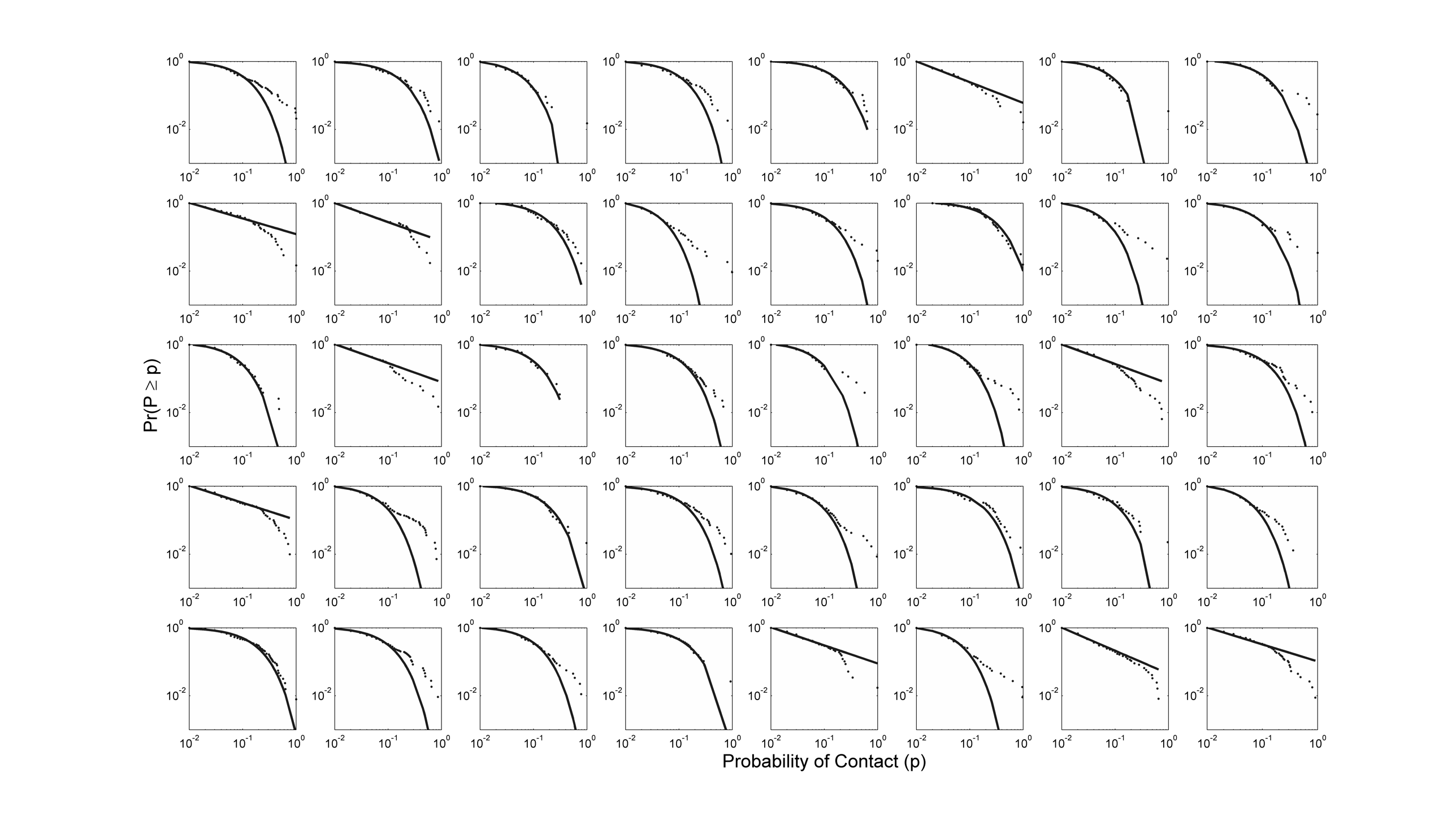

Supplement: Figure S1 — Individual Fits of Exponential and Power Functions on the Distribution of Contact Probability. Both exponential and power functions were fitted to the individual distributions of contact probability across the members of the person's social network. For each participant the distribution and the best-fitting function is shown on a log-log scale. For 31 of the 40 participants, an exponential function (curved relationship) yielded a better fit than a power function (linear relationship). (TIF) [file pone.0086081.s001.tif]

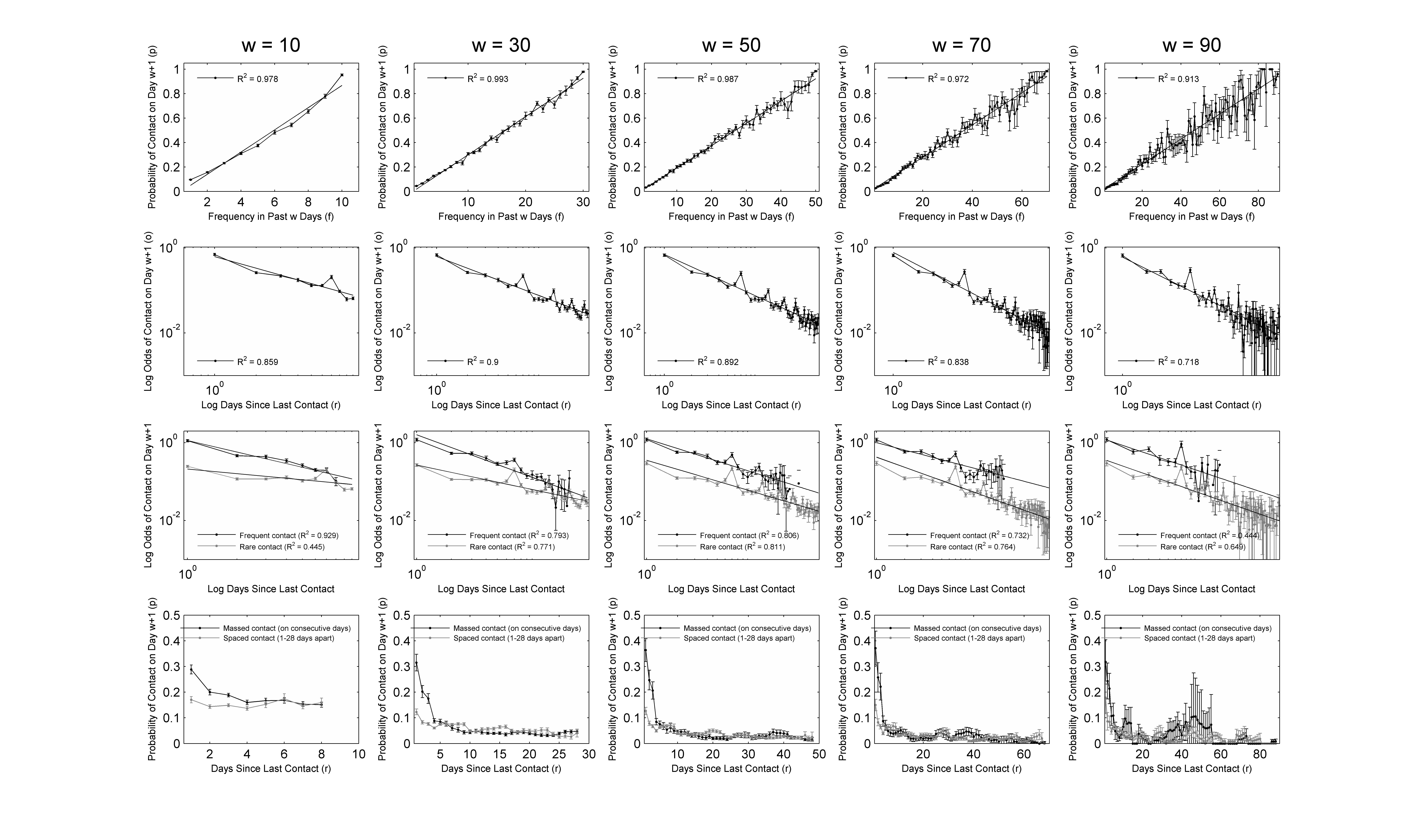

Supplement: Figure S2 — Frequency, Recency, and Spacing Effects on Contact Probability for Window Sizes w = 10, 30, 50, 70, and 90. In the recency analysis separating frequent and rare contacts, frequent contacts were defined as those occurring on at least one fifth of the days in the time window (that is, on ≥w/5 days); rare contacts were defined as those occurring less frequently than that. R2 values in the second and third rows were computed on the log-transformed data. (TIF) [file pone.0086081.s002.tif]

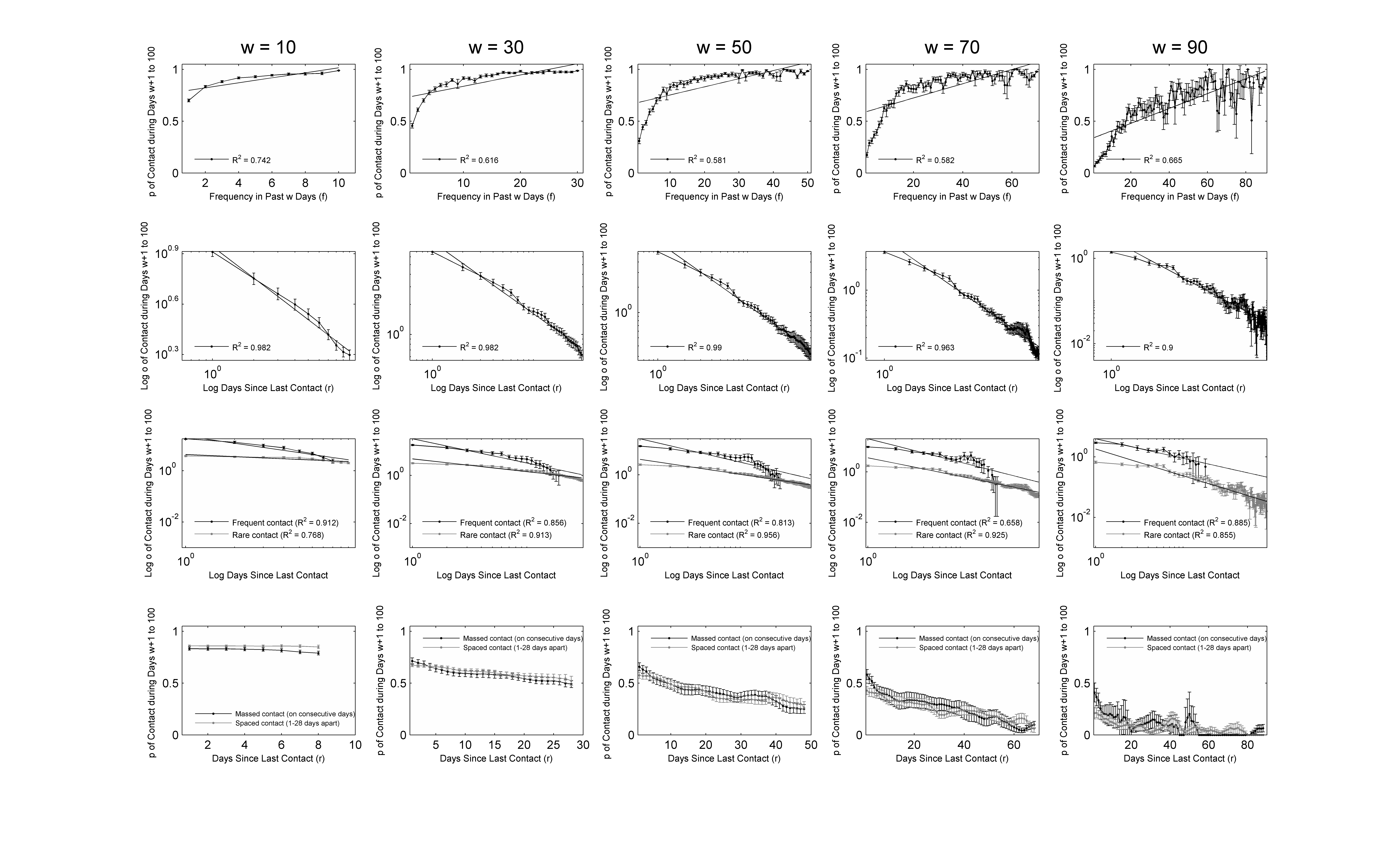

Supplement: Figure S3 — Frequency, Recency, and Spacing Effects on Presence Probability for Window Sizes w = 10, 30, 50, 70, and 90. Presence probability is defined as the probability that there is at least one contact during days w+1 to 100. In the recency analysis separating frequent and rare contacts, frequent contacts were defined as those occurring on at least one fifth of the days in the time window (that is, on ≥w/5 days); rare contacts were defined as those occurring less frequently than that. R2 values in the second and third rows were computed on the log-transformed data. R2 values in the second and third rows were computed on the log-transformed data. (TIF) [file pone.0086081.s003.tif]
