# Supplementary material for: We'll Meet Again: Revealing Distributional and Temporal Patterns of Social Contact
Source: PLoS One. 2014 Jan 27;9(1):e86081. doi: 10.1371/journal.pone.0086081 (PMC3903503; doi:10.1371/journal.pone.0086081)
Supplement: Table S1 — Regression functions for frequency and recency effects when using window sizes 10, 30, 50, 70, and 90. (DOCX) [file pone.0086081.s005.docx]

**Table S1.** Regression functions for frequency and recency effects when using window sizes 10, 30, 50, 70, and 90.

|  | Predictor | | | |
| --- | --- | --- | --- | --- |
| Window size (*w*) | Frequency | | Recency | |
|  | Function | *R^2^* | Function | *R^2^* |
| 10 | –0.042 + 0.091 *f* | 0.978 | 0.624 *r*^–0.91^ | 0.859 |
| 30 | –0.014 + 0.031 *f* | 0.993 | 0.626 *r*^–0.91^ | 0.900 |
| 50 | 0.010 + 0.018 *f* | 0.987 | 0.699 *r*^–0.96^ | 0.892 |
| 70 | 0.015 + 0.013 *f* | 0.972 | 0.785 *r*^–1.03^ | 0.838 |
| 90 | 0.019 + 0.010 *f* | 0.913 | 0.605 *r*^–0.94^ | 0.718 |
