# Supplementary material for: We'll Meet Again: Revealing Distributional and Temporal Patterns of Social Contact
Source: PLoS One. 2014 Jan 27;9(1):e86081. doi: 10.1371/journal.pone.0086081 (PMC3903503; doi:10.1371/journal.pone.0086081)
Supplement: Table S2 — As a focus on the probability of contact on a particular day (i.e., w +1) might be overly restrictive, we also examined how the frequency and recency of contact in the time window of size w relates to the probability of another contact on at least one of the remaining days (i.e., day w +1 to day 100), to which we refer to as ‘presence probability’. (For the effect of the spacing of contacts on presence probability, see Figure S3). (DOCX) [file pone.0086081.s006.docx]

**Table S2.** As a focus on the probability of contact on a particular day (i.e., *w* + 1) might be overly restrictive, we also examined how the frequency and recency of contact in the time window of size *w* relates to the probability of another contact on at least one of the remaining days (i.e., day *w* + 1 to day 100), to which we refer to as ‘presence probability’. (For the effect of the spacing of contacts on presence probability, see Figure S3).

|  | Predictor | | | |
| --- | --- | --- | --- | --- |
| Window size (*w*) | Frequency | | Recency | |
|  | Function | *R^2^* | Function | *R^2^* |
| 10 | 0.772 + 0.024 *f* | 0.739 | 8.773 *r*^–0.624^ | 0.982 |
| 30 | 0.727 + 0.011 *f* | 0.621 | 8.502 *r*^–0.722^ | 0.983 |
| 50 | 0.674 + 0.008 *f* | 0.578 | 7.077 *r*^–0.764^ | 0.988 |
| 70 | 0.586 + 0.007 *f* | 0.581 | 5.625 *r*^–0.854^ | 0.965 |
| 90 | 0.336 + 0.007 *f* | 0.660 | 2.789 *r*^–0.998^ | 0.903 |
