# Supplementary material for: We'll Meet Again: Revealing Distributional and Temporal Patterns of Social Contact
Source: PLoS One. 2014 Jan 27;9(1):e86081. doi: 10.1371/journal.pone.0086081 (PMC3903503; doi:10.1371/journal.pone.0086081)
Supplement: Table S3 — Regression functions for frequency and recency effects separately for self-initiated and received contacts. (DOCX) [file pone.0086081.s007.docx]

**Table S3.** Regression functions for frequency and recency effects separately for self-initiated and received contacts.

|  | Predictor | | | |
| --- | --- | --- | --- | --- |
| Direction of contact | Frequency | | Recency | |
|  | Function | *R^2^* | Function | *R^2^* |
| Self-initiated | –0.013 + 0.031 *f* | 0.995 | 0.366 *r*^–0.775^ | 0.867 |
| Received | 0.003 + 0.030 *f* | 0.982 | 0.353 *r*^–0.861^ | 0.835 |
