# Supplementary material for: We'll Meet Again: Revealing Distributional and Temporal Patterns of Social Contact
Source: PLoS One. 2014 Jan 27;9(1):e86081. doi: 10.1371/journal.pone.0086081 (PMC3903503; doi:10.1371/journal.pone.0086081)
Supplement: Table S4 — Regression functions for frequency and recency effects on contact medium. (DOCX) [file pone.0086081.s008.docx]

**Table S4.** Regression functions for frequency and recency effects on contact medium.

|  | Predictor | | | |
| --- | --- | --- | --- | --- |
| Contact medium | Frequency | | Recency | |
|  | Function | *R^2^* | Function | *R^2^* |
| Face-to-face | –0.006 + 0.029 *f* | 0.995 | 0.373 *r*^–0.826^ | 0.799 |
| Email | –0.006 + 0.023 *f* | 0.729 | 0.139 *r*^–0.559^ | 0.860 |
| Phone | –0.008 + 0.029 *f* | 0.911 | 0.265 *r*^–0.725^ | 0.865 |
| Other | –0.008 + 0.019 *f* | 0.482 | 0.140 *r*^–0.592^ | 0.803 |
